# Peripheral Oxygen Saturation Targets and Hyperoxemia in Critical Care: Influence of pH, F_i_O_2_, and Respiratory Failure

**DOI:** 10.3390/antiox15020235

**Published:** 2026-02-11

**Authors:** Marcos Delgado, Robert Fritze, Matthias P. Hilty, Michael Krauthammer, Reto A. Schuepbach, Christoph Ganter, Jan Bartussek

**Affiliations:** 1 Institute of Intensive Care Medicine, University Hospital Zurich, University of Zurich, Raemistrasse 100, CH-8091 Zurich, Switzerland; matthias.hilty@usz.ch (M.P.H.); reto.schuepbach@usz.ch (R.A.S.); christoph.ganter@usz.ch (C.G.); jan.bartussek@usz.ch (J.B.); 2 Department of Anesthesiology and Intensive Care Medicine, University Hospital Krems–NOE LGA, Karl Landsteiner University, Mitterweg 10, 3500 Krems, Austria; robert.fritze@krems.lknoe.at; 3 Department for Quantitative Biomedicine, University of Zurich, Raemistrasse 100, CH-8091 Zurich, Switzerland; michael.krauthammer@usz.ch

**Keywords:** oxidative stress, blood pH, oxygen therapy, intensive care, respiratory failure, hyperoxemia

## Abstract

Oxygen therapy is a cornerstone in the treatment of critically ill patients. However, excess oxygen administration may promote oxidative cellular injury once hemoglobin is fully saturated and additional oxygen remains dissolved, enhancing reactive oxygen species formation. The combined impact of oxygen administration, pH, and respiratory failure on hyperoxemia across saturation ranges is not well understood. We conducted a retrospective study at a tertiary center to assess how these factors modify hyperoxemia frequency in adult ICU patients. Continuous S_p_O_2_ measurements were aligned with arterial blood gases (P_a_O_2_, pH, F_i_O_2_), and hyperoxemia was evaluated using predefined P_a_O_2_ thresholds (>120 mmHg and >150 mmHg). Among 21,406 patients with 717,064 paired measurements, prolonged hyperoxemia occurred in over half of mechanically ventilated patients, most commonly in those without or with mild-to-moderate respiratory failure. Acidotic states were associated with higher P_a_O_2_ values at comparable S_p_O_2_ levels, consistent with a rightward shift in the oxygen–hemoglobin dissociation curve. S_p_O_2_ values ≥ 98% were consistently associated with hyperoxemia, whereas 96–97% generally corresponded to P_a_O_2_ within physiological ranges. Higher F_i_O_2_ markedly increased hyperoxemia probability, allowing derivation of pH-stratified F_i_O_2_ exposure limits. Our findings highlight the importance of individualized oxygen therapy, considering pH and respiratory failure phenotype to guide safer oxygen management.

## 1. Introduction

Oxygen therapy is fundamental in critical care and perioperative medicine to prevent insufficient oxygen levels (hypoxemia) and support organ function. However, supraphysiological arterial oxygen tensions (hyperoxemia) are frequently observed in routine clinical practice. While hypoxemia is carefully avoided, elevated arterial oxygen tension levels (P_a_O_2_) are often tolerated, despite accumulating evidence that hyperoxemia represents an undesirable exposure in critically ill patients [1,2,3]. From a physiological perspective, once hemoglobin is fully saturated, additional oxygen is dissolved in plasma without further benefit for oxygen delivery, while experimental and clinical studies have linked excessive oxygen exposure to oxidative pathways and cellular injury [4,5]. Consequently, current guidelines recommend oxygen strategies that avoid both hypoxemia and unnecessary hyperoxemia [6,7,8]. Nevertheless, in daily practice, liberal oxygen administration remains frequent [1].

Peripheral oxygen saturation (S_p_O_2_) is widely used for continuous, non-invasive monitoring of oxygenation, but its ability to reflect arterial oxygen tension is limited at high saturation levels. Due to the plateau of the oxygen–hemoglobin dissociation curve, substantial increases in P_a_O_2_ may occur with little or no change in S_p_O_2_ once saturation exceeds approximately 95–97% [9,10]. This dissociation is further modulated by physiological factors such as temperature, 2,3-diphosphoglycerate, acid-base status, and carbon dioxide tension, all of which influence hemoglobin-oxygen affinity and thereby alter the relationship between S_p_O_2_ and P_a_O_2_. While temperature effects are generally relevant only at extreme values, the role of 2,3-Diphosphoglycerate, a glycolytic byproduct, is poorly understood and subject to variation due to factors such as diet, physical activity, chronic disease, and hypoxic conditions. Additionally, its routine clinical assessment is limited [11]. Consequently, acid-base status and carbon dioxide tension remain the most practical and routinely monitored modulators in clinical settings [12]. As a result, clinically acceptable S_p_O_2_ targets may conceal supraphysiological P_a_O_2_ levels, particularly in the presence of acidosis or altered respiratory physiology.

Although the determinants of oxygen transport are well established, real-world data quantifying how frequently hyperoxemia occurs across commonly used S_p_O_2_ targets, and how this probability is modified by blood pH, inspired oxygen fraction (F_i_O_2_), and severity of respiratory failure, remain limited. Prior studies have largely focused on outcome associations of different oxygenation strategies [13,14,15,16], but have not systematically characterized the physiological exposure to supraphysiological P_a_O_2_ across saturation targets in routine clinical practice. It remains unclear how diffusion limitation in advanced respiratory failure influences the likelihood of hyperoxemia at given S_p_O_2_ and F_i_O_2_ levels.

We therefore performed a large retrospective observational analysis in ICU patients to characterize the relationship between S_p_O_2_, P_a_O_2_, pH, F_i_O_2_, and respiratory failure severity using high-resolution clinical data. Our primary objective was to quantify the probability of exceeding predefined P_a_O_2_ thresholds across S_p_O_2_ ranges and to assess how acid-base status and respiratory failure modify this relationship. While overall exposure patterns were described for the full cohort, detailed analyses focused on mechanically ventilated patients, in whom hyperoxemia was most prevalent. By providing a physiologically grounded, exposure-based assessment of oxygenation patterns, this study aims to inform more precise interpretation of S_p_O_2_ targets in critically ill patients.

## 2. Materials and Methods

### 2.1. Study Design and Setting

We conducted a retrospective cohort study at the University Hospital Zurich, a tertiary care center, between November 2017 and December 2022. Ethical approval was granted by the cantonal ethics committee of Zurich (BASEC No. 2019-02015). Patients admitted to the intensive care unit (ICU), including both medical and surgical patients requiring ICU-level saturation monitoring, were screened.

### 2.2. Study Population

Adults (≥18 years) were eligible if they did not object further use of health data, had continuous S_p_O_2_ monitoring and at least one matched arterial blood gas measurement. Patients receiving extracorporeal membrane oxygenation or with implausible physiologic values were excluded. For reference, patients never treated with oxygen were included to assess age-related changes in oxygenation. The final cohort comprised 21,406 patients.

### 2.3. Data Collection

Clinical data were extracted from the institutional Patient Data Management System, which records vital signs and laboratory results at one-minute intervals. Collected variables included S_p_O_2_, P_a_O_2_, arterial carbon dioxide tension (P_a_CO_2_), arterial pH, F_i_O_2_, arterial hemoglobin saturation (S_a_O_2_), demographics, comorbidities, and severity of Acute Respiratory Distress Syndrome (ARDS). S_p_O_2_ was continuously recorded, whereas arterial blood gas parameters were available at discrete sampling time points.

Arterial blood gas measurements were aligned with the median S_p_O_2_ and F_i_O_2_ recorded in the preceding five minutes. Details on signal alignment and quality control are provided in the online data supplement.

### 2.4. Definitions

Hyperoxemia was defined using predefined P_a_O_2_ thresholds (>120 mmHg and >150 mmHg), chosen to reflect commonly used supraphysiological ranges in the critical care literature [2,17,18]. Arterial blood gases were stratified by pH (normal 7.35–7.45, acidotic < 7.35, alkalotic > 7.45) and P_a_CO_2_ (hypocapnic < 35 mmHg, normocapnic 35–45 mmHg, hypercapnic > 45 mmHg). The P_a_O_2_/F_i_O_2_ ratio was calculated, and acute respiratory distress syndrome severity was defined according to the Berlin criteria [19]. ARDS severity was used to describe the degree respiratory failure and diffusion impairment, rather than as a marker of overall disease severity or outcome.

### 2.5. Outcomes

The primary outcome was the frequency and probability of exceeding predefined P_a_O_2_ thresholds across S_p_O_2_ ranges. Secondary outcomes included the modifying effects of pH, F_i_O_2_, age, and severity of acute respiratory distress syndrome.

### 2.6. Stratification and Subgroup Analyses

In our study, patients were categorized based on their oxygen treatment status, with three primary groups: mechanically ventilated patients (invasive and non-invasive), patients receiving oxygen therapy (nasal cannula, high-flow nasal cannula, face mask), and patients without supplemental oxygen. These groups were not mutually exclusive, as each patient had multiple measurements across different time points, and their treatment modality could change over time. This means that a patient could, at different times, fall into more than one group based on the oxygen therapy or ventilation they received at that specific time. In contrast, for the age-related analysis, we included only patients who were never treated with oxygen.

The numbers of paired measurements and corresponding patients in each group were as follows:

- mechanically ventilation: 443,225 paired measurements from 17,439 patients
- oxygen therapy: 208,136 measurements from 15,439 patients
- no supplemental oxygen: 69,227 measurements from 12,332 patients
- never treated with oxygen: 4131 measurements from 1140 patients (age-related control group)

Because hyperoxemia was uncommon across the non-mechanically ventilated groups, detailed stratified analyses were restricted to mechanically ventilated patients to ensure sufficient exposure prevalence.

### 2.7. Statistical Analysis

Clinical characteristics were summarized descriptively. Hyperoxemia prevalence was assessed using patient-level summary statistics during the respective treatment period. Distributions of P_a_O_2_ and F_i_O_2_ were analyzed using histograms and cumulative distribution functions. In mechanically ventilated patients, where hyperoxemia was sufficiently prevalent to allow stable probability estimates, the joint relationship between oxygen exposure and oxygen saturation was analyzed using F_i_O_2_ and S_p_O_2_, and contour plots stratified by pH category were used to display absolute probabilities of exceeding predefined P_a_O_2_ thresholds. Analyses were performed with MATLAB R2024b.

## 3. Results

### 3.1. Patient Characteristics

The cohort included 21,406 adults with 717,064 paired S_p_O_2_–arterial blood gas measurements (see Table S1 in the Supplementary Materials). Most patients were aged 60–80 years, and 62% were male. Among the medical and surgical ICU patients included in the study, cardiovascular disease was the most common primary admission diagnosis, followed by malignancy and neurological disorders. Acute respiratory distress syndrome was present in 57% of patients, with 15% classified as mild, 21% as moderate, and 21% as severe (Table 1).

In the subgroup of patients who never received oxygen therapy during their stay (*n* = 1140), the mean P_a_O_2_ was 103 mmHg. In these patients, P_a_O_2_ values declined slightly with age, but the reduction was less pronounced than predicted by Sorbini’s formula [20] (see Figure S3 in the Supplementary Materials).

### 3.2. Prevalence of Hyperoxemia

**Figure 1:**
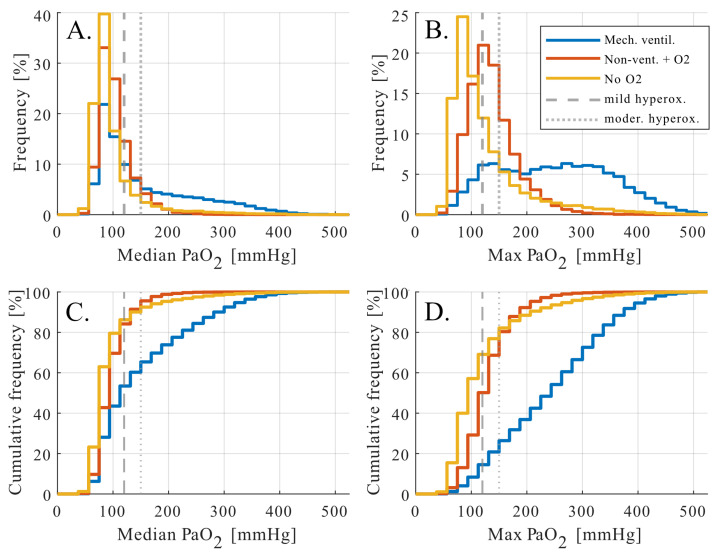
Hyperoxemia prevalence among ICU patients by oxygen delivery modality. Distribution of arterial oxygen tension (P_a_O_2_) by oxygen delivery modality. Histograms (**top**) and cumulative distributions (**bottom**) are shown for patients without oxygen (orange), patients receiving oxygen (red), and mechanically ventilated patients (blue). Dashed and dotted lines indicate mild (P_a_O_2_ > 120 mmHg) and moderate (P_a_O_2_ > 150 mmHg) hyperoxemia thresholds. Hyperoxemia was most frequent in mechanically ventilated patients. Different treatment groups: (**A**) frequency distribution of median P_a_O_2_, (**B**) frequency distribution of maximum P_a_O_2_, (**C**) cumulative frequency distribution of median P_a_O_2_, and (**D**) cumulative frequency distribution of maximum P_a_O_2_.

To distinguish between transient oxygen peaks and sustained exposure, hyperoxemia prevalence was assessed using patient-level summary statistics, specifically the median and maximum P_a_O_2_ observed during the treatment period (Figure 1). Median P_a_O_2_ values characterize sustained oxygen exposure, whereas maximum P_a_O_2_ values capture short-lived peaks, often occurring during procedures or acute clinical events.

When summarized using median P_a_O_2_ values, patients without supplemental oxygen and those receiving oxygen showed narrow distributions centered within physiological ranges, with only a small fraction exceeding the predefined hyperoxemia thresholds. In contrast, mechanically ventilated patients exhibited a markedly right-shifted distribution. More than half of mechanically ventilated patients had a median P_a_O_2_ above 120 mmHg, indicating sustained exposure to supraphysiological arterial oxygen tensions over a substantial portion of their monitored course (Figure 1A,C). Approximately one third exceeded a median P_a_O_2_ above 150 mmHg, reflecting prolonged moderate hyperoxemia at the patient level.

Analysis of maximum P_a_O_2_ values revealed a distinct and complementary pattern. While non ventilated patients with oxygen support rarely exceeded moderate hyperoxemia thresholds, mechanically ventilated patients frequently experienced brief but pronounced oxygen peaks (Figure 1B,D). Approximately 80% of mechanically ventilated patients reached maximum P_a_O_2_ values above 150 mmHg, with rare extreme values extending beyond 400 mmHg. In contrast, supraphysiological P_a_O_2_ values observed in patients without documented oxygen therapy occurred almost exclusively as isolated maxima and likely reflect technical or documentation-related artifacts rather than sustained physiological exposure. These values did not influence patient-level median P_a_O_2_ and were not considered further (considered implausible physiologic values).

### 3.3. F_i_O_2_ Exposure Patterns

**Figure 2:**
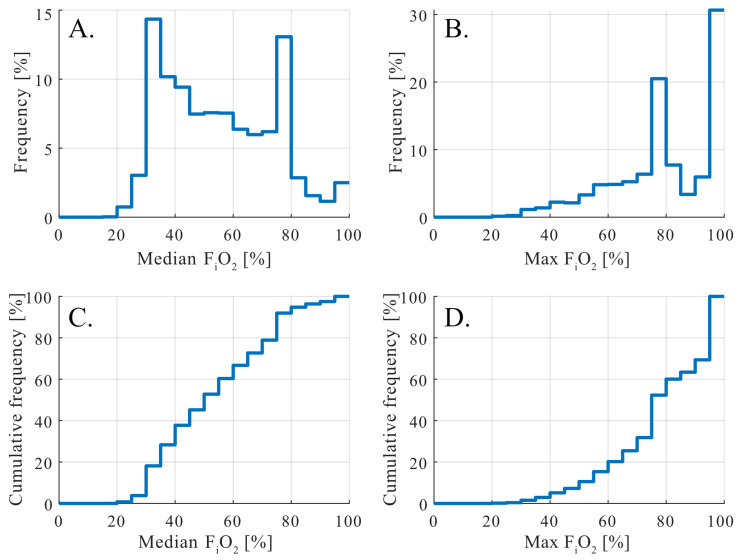
Distribution of inspired oxygen fraction according to management strategy. Inspired oxygen fraction (F_i_O_2_) distributions in mechanically ventilated patients. Histograms (**top**) and cumulative distributions (**bottom**) of median and maximum F_i_O_2_ per patient. Peaks at 35–40% and 80–90% reflect common management strategies. Over half of patients reached F_i_O_2_ > 80% at least once, and one-third received F_i_O_2_ of 100%. (**A**) frequency distribution of median F_i_O_2_, (**B**) frequency distribution of maximum F_i_O_2_, (**C**) cumulative frequency distribution of median F_i_O_2_, and (**D**) cumulative frequency distribution of maximum F_i_O_2_.

Inspired oxygen fraction distributions in mechanically ventilated patients demonstrated distinct exposure patterns when summarized at the patient level (Figure 2). Median F_i_O_2_ values showed a bimodal distribution with peaks at approximately 35–40% and 80–90%, indicating two commonly employed oxygenation strategies during mechanical ventilation (Figure 2A,C). While the lower peak likely reflects routine maintenance oxygenation, the higher peak suggests sustained use of elevated F_i_O_2_ levels in a substantial proportion of patients.

Analysis of maximum F_i_O_2_ values revealed an even more pronounced right-skewed distribution (Figure 2B,D). More than half of mechanically ventilated patients were exposed to F_i_O_2_ levels above 80% at least once during their ICU stay, and approximately one third reached a maximum F_i_O_2_ of 100%. These high F_i_O_2_ values most plausibly reflect short-term escalation during acute clinical events, procedures, or preoxygenation, rather than continuous baseline therapy.

The median and maximum F_i_O_2_ distributions highlight a clinically relevant separation between prolonged oxygen exposure and transient oxygen peaks, analogous to the patterns observed for P_a_O_2_. Importantly, these findings demonstrate that exposure to high inspired oxygen fractions is common in routine ICU practice, providing a physiological context for subsequent analyses examining how F_i_O_2_ interacts with S_p_O_2_ and blood pH to determine the probability of supraphysiological arterial oxygen tensions.

### 3.4. Effect of F_i_O_2_ Across S_p_O_2_ Target Ranges

**Figure 3:**
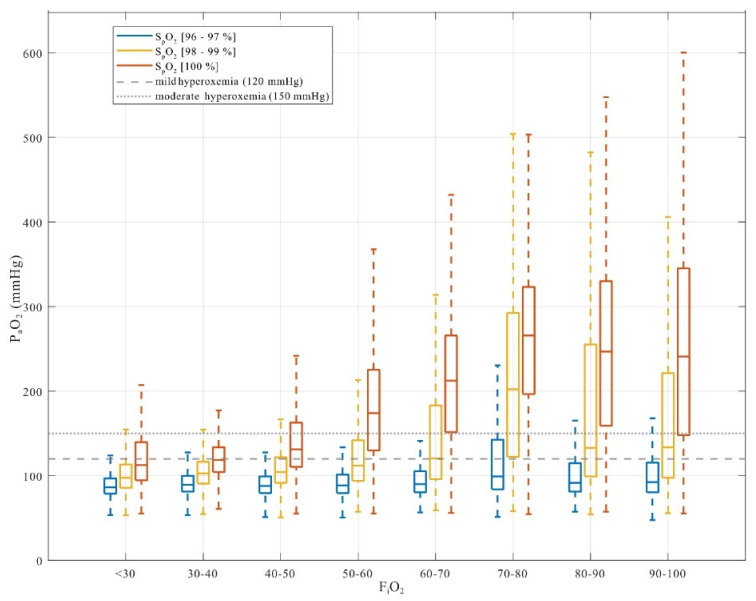
Comparison of F_i_O_2_ levels, P_a_O_2_ achieved, and resulting S_p_O_2_ across different patient groups. Bars represent S_p_O_2_ targets of 96–97% (blue), 98–99% (yellow), and 100% (red). Horizontal lines indicate mild (P_a_O_2_ > 120 mmHg) and moderate (P_a_O_2_ > 150 mmHg) hyperoxemia thresholds. Saturations ≥ 98% were consistently associated with hyperoxemia.

Across mechanically ventilated patients, the relationship between inspired oxygen fraction and arterial oxygen tension differed markedly by S_p_O_2_ target range (Figure 3). When S_p_O_2_ was maintained between 96–97%, P_a_O_2_ values remained largely within physiological limits across a wide range of F_i_O_2_ levels, with only a small proportion exceeding the predefined hyperoxemia thresholds. Even at higher F_i_O_2_ levels, P_a_O_2_ distributions in this saturation range showed limited rightward extension.

In contrast, S_p_O_2_ targets of 98–99% were associated with a pronounced upward shift in P_a_O_2_ distributions that became progressively more evident with increasing F_i_O_2_. At these saturation levels, P_a_O_2_ frequently exceeded the mild hyperoxemia threshold and, at moderate to high F_i_O_2_, often surpassed the moderate threshold. This effect was further amplified at S_p_O_2_ of 100%, where P_a_O_2_ values increased steeply and displayed substantial variability across all but the lowest F_i_O_2_ categories.

### 3.5. Influence of pH on the S_p_O_2_-P_a_O_2_ Relationship

**Figure 4:**
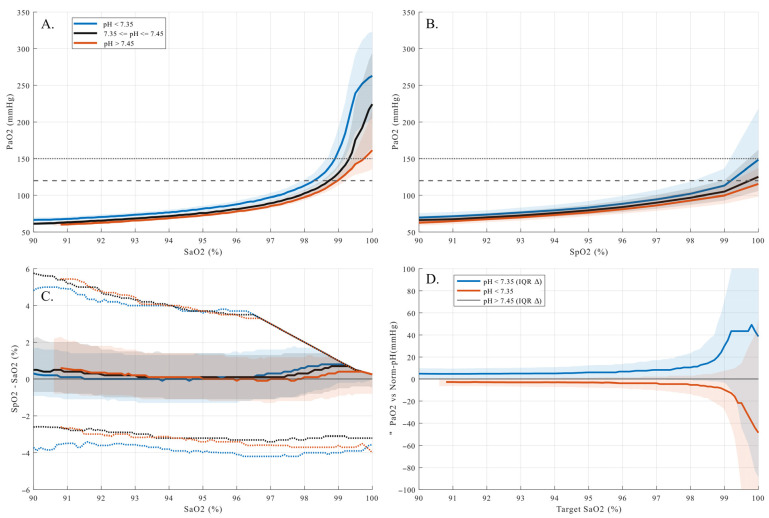
pH-dependent arterial oxygen exposure and pulse oximetry behavior across the oxygen–hemoglobin saturation range. Panels show the relationship between arterial oxygen tension (P_a_O_2_, mmHg) and oxygen saturation, stratified by arterial pH (<7.35, 7.35–7.45, >7.45). (**A**): P_a_O_2_ as a function of S_a_O_2_ (0.1% bins). (**B**): P_a_O_2_ as a function of S_p_O_2_ (1% bins). Solid lines indicate the median, shaded areas the interquartile range (25th–75th percentile). Dashed horizontal lines denote used hyperoxemia thresholds (120 and 150 mmHg). (**C**): Difference between S_p_O_2_ and S_a_O_2_ across S_a_O_2_, illustrating pH-dependent pulse oximetry bias; in addition to the median and interquartile range, dotted lines indicate the 5th and 95th percentiles to visualize tail dispersion. (**D**): Difference in P_a_O_2_ relative to the normophysiological pH group (7.35–7.45) at fixed S_a_O_2_, demonstrating increasing pH-dependent divergence in the saturation plateau.

Acid–base status exerted a strong and systematic influence on the relationship between arterial oxygen saturation and arterial oxygen tension (Figure 3). When P_a_O_2_ was plotted against S_a_O_2_ (Figure 4A), a clear rightward displacement of the oxygen–hemoglobin dissociation curve was observed under acidotic conditions, whereas alkalosis was associated with lower P_a_O_2_ values at comparable saturation levels.

At a fixed S_a_O_2_ of 99%, median P_a_O_2_ differed markedly across pH groups (Kruskal–Wallis *p* < 10^−300^). Acidotic patients (pH < 7.35) exhibited a median P_a_O_2_ of 154.5 mmHg (interquartile range [IQR] 132.0–198.8 mmHg; n = 23,159), compared with 126.0 mmHg (IQR 115.5–141.8 mmHg; n = 73,509) in the normophysiological pH group and 117.0 mmHg (IQR 108.0–128.3 mmHg; n = 28,901) under alkalotic conditions. These differences became even more pronounced at S_a_O_2_ = 100% (Kruskal–Wallis *p* < 10^−300^), where median P_a_O_2_ reached 258.0 mmHg (IQR 201.0–317.3 mmHg; *n* = 8408) in acidotic patients, compared with 220.5 mmHg (IQR 160.5–291.0 mmHg; *n* = 25,341) in the normophysiological group and 164.3 mmHg (IQR 135.8–245.3 mmHg; *n* = 8802) in alkalotic patients. Thus, identical arterial oxygen saturations were associated with substantially different, and increasingly supraphysiological, P_a_O_2_ values depending on pH, particularly within the saturation plateau.

When P_a_O_2_ was referenced to S_p_O_2_ (Figure 4B), similar directional trends across pH groups were present, but the separation between groups appeared attenuated and the overall dispersion visually compressed. This apparent smoothing is explained by the behavior of pulse oximetry bias illustrated in Figure 4C. Although the median difference between S_p_O_2_ and S_a_O_2_ remained close to zero across the saturation range, both the interquartile range and the tail dispersion widened progressively with increasing S_a_O_2_, most prominently under acidotic conditions. Consequently, at high saturation levels, S_p_O_2_ increasingly masked substantial variability and elevation in P_a_O_2_, limiting its ability to reliably reflect arterial oxygen tension.

This effect is summarized in Figure 4D, which shows the difference in P_a_O_2_ relative to the normophysiological pH group across the saturation range. The pH-dependent divergence increased sharply within the saturation plateau, highlighting that, small differences in measured saturation corresponded to large and clinically relevant differences in arterial oxygen tension.

Detailed boxplot representations and group-wise distributions at fixed S_a_O_2_ and S_p_O_2_ levels are provided in the Supplementary Material (Figure S4), supporting the robustness of these findings across the full range of observed data.

### 3.6. Absolute Probability Estimates

**Figure 5:**
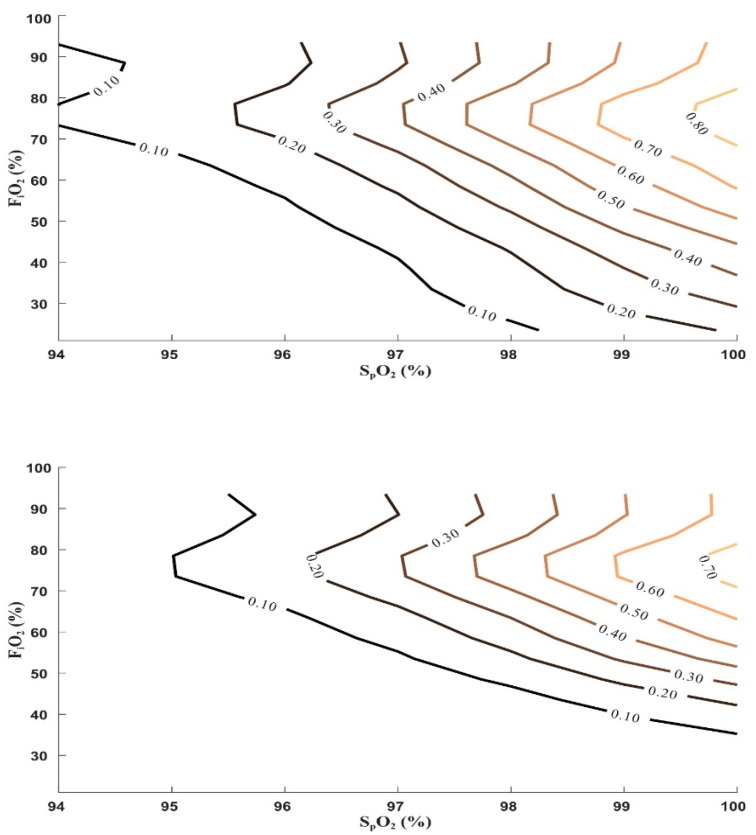
Contour plot of absolute probability for mild and moderate hyperoxemia by normal pH. Absolute probability increased progressively with higher F_i_O_2_ and S_p_O_2_, with contour lines indicating probabilities in 10% increments. At F_i_O_2_ >0.8, the risk plateaued or declined, reflecting the contribution of severe ARDS patients who require high F_i_O_2_ but seldom reach hyperoxemia due to diffusion limitation. Color changes from darker to lighter shades represent increasing absolute probability of exceeding the thresholds for mild and moderate hyperoxemia.

Contour plots were used to visualize the joint relationship between S_p_O_2_, F_i_O_2_, and the probability of exceeding predefined P_a_O_2_ thresholds in mechanically ventilated patients (Figure 5). Across the examined range, probability increased progressively with both higher S_p_O_2_ and higher F_i_O_2_; however, the increase was non-linear and occurred over relatively narrow S_p_O_2_ intervals. In particular, probability contours showed a steep transition beginning at S_p_O_2_ values of approximately 97–98%, beyond which small increments in saturation were associated with large increases in the probability of hyperoxemia across a wide range of F_i_O_2_ levels.

The contour plots further delineated contiguous regions of low and high probability. At lower S_p_O_2_ targets, extended areas remained below a 10% probability threshold even at moderate F_i_O_2_ levels, whereas at higher S_p_O_2_ targets, probability increased rapidly and exceeded 30–50% across commonly used F_i_O_2_ ranges. These patterns were consistent across probability thresholds depicted in 10% increments.

Stratified contour plots demonstrated that these probability surfaces were systematically shifted by blood pH (Figures S1 and S2 in the Supplementary Materials). Compared with normal pH, acidotic conditions were associated with leftward and upward displacement of probability contours, indicating higher probabilities of hyperoxemia at lower S_p_O_2_ and F_i_O_2_ combinations. In contrast, alkalotic conditions shifted contours toward higher S_p_O_2_ and F_i_O_2_ values. Based on these contour-derived probability surfaces, Table 2 summarizes F_i_O_2_ limits stratified by S_p_O_2_ and pH corresponding to an absolute probability of hyperoxemia below 10%.

**Figure 6:**
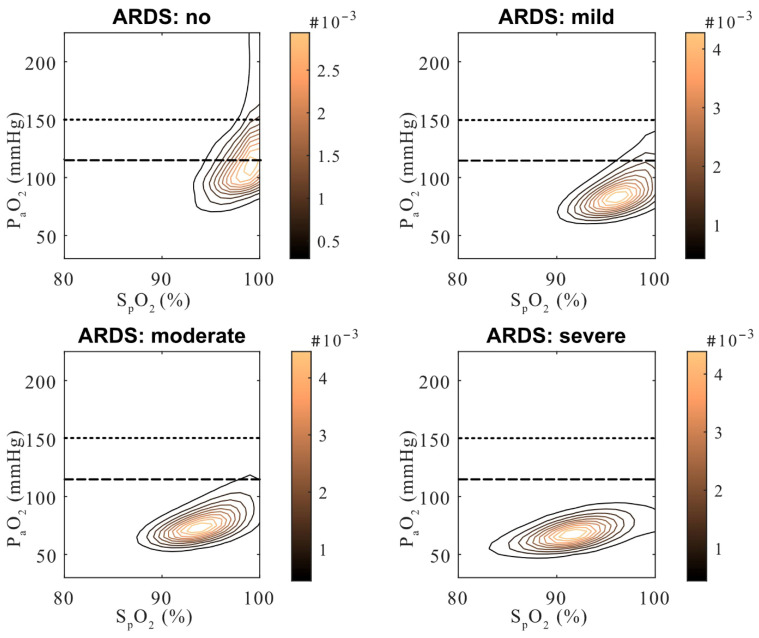
P_a_O_2_-S_p_O_2_ density distributions by ARDS severity. Kernel density plots show P_a_O_2_-S_p_O_2_ combinations across ARDS categories. Patients without ARDS or with mild ARDS most frequently exceeded hyperoxemia thresholds, while severe ARDS rarely did so due to diffusion limitation. Horizontal lines indicate mild (P_a_O_2_ > 120 mmHg) and moderate (P_a_O_2_ > 150 mmHg) hyperoxemia thresholds. Dashed horizontal lines denote used hyperoxemia thresholds (120 and 150 mmHg).

At high inspired oxygen fractions (F_i_O_2_ > 0.8), the probability of hyperoxemia did not continue to increase monotonically but instead plateaued or declined in the overall cohort. To clarify this pattern, P_a_O_2_-S_p_O_2_ density distributions were examined across strata of ARDS severity (Figure 6).

Patients without ARDS and those with mild ARDS most frequently exceeded the predefined hyperoxemia thresholds, with density distributions extending into supraphysiological P_a_O_2_ ranges at high S_p_O_2_ values. In contrast, patients with severe ARDS demonstrated markedly compressed P_a_O_2_ distributions despite high S_p_O_2_ and F_i_O_2_ exposure, rarely reaching P_a_O_2_ levels associated with hyperoxemia. Patients with moderate ARDS showed intermediate patterns between these extremes.

## 4. Discussion

This large retrospective analysis of more than 700,000 paired S_p_O_2_–arterial blood gas measurements from over 21,000 critically medical and surgical patients provides a detailed characterization of arterial oxygen exposure in routine clinical practice. Rather than evaluating clinical outcomes, our study focuses on the physiological conditions under which supraphysiological arterial oxygen tensions occur and how commonly used saturation targets interact with inspired oxygen fraction, acid-base status, and respiratory failure severity grades. Several findings emerge that extend prior observations and help contextualize current oxygenation practices.

A first key observation is the strong association between high peripheral oxygen saturation targets and the probability of hyperoxemia. Across a wide range of clinical conditions, S_p_O_2_ values ≥98% were consistently associated with supraphysiological P_a_O_2_ levels, whereas a target range of 96–97% was generally associated with P_a_O_2_ values below commonly used hyperoxemia thresholds (Figure 3). Although this saturation range is widely considered “normal” in clinical practice, our data demonstrate that small differences within the upper plateau of the oxygen–hemoglobin dissociation curve translate into substantial differences in arterial oxygen exposure. This finding does not contradict established physiological principles but quantifies their impact under real-world conditions and across a large and heterogeneous ICU population.

Inspired oxygen fraction emerged as a major determinant of arterial oxygen exposure, not only through its absolute level but also through the duration and variability of exposure. The bimodal F_i_O_2_ distribution observed in mechanically ventilated patients, with peaks around 35–40% and 80–90%, reflects common management strategies, including routine oxygen supplementation and short-term escalation during procedures or acute deterioration (Figure 2). Importantly, our analysis distinguishes between sustained oxygen exposure and transient F_i_O_2_ peaks, showing that hyperoxemia is not limited to brief extremes but frequently reflects prolonged exposure at commonly applied F_i_O_2_ levels. These findings emphasize that arterial hyperoxemia can occur even in the absence of exceptionally high F_i_O_2_ when saturation targets are set near the upper plateau of hemoglobin saturation.

Blood pH acted as a systematic physiological modifier of arterial oxygen exposure. Acidosis was associated with higher P_a_O_2_ values at equivalent S_p_O_2_ or S_a_O_2_ levels, consistent with rightward shifts in the oxygen–hemoglobin dissociation curve. As a result, hyperoxemia occurred at lower saturation targets under acidotic conditions compared with normal or alkalotic pH. This effect was not confined to extreme values but was evident across commonly encountered clinical ranges (Figure 4). While the influence of pH on hemoglobin-oxygen affinity is well established, our findings demonstrate how this interaction translates into differences in arterial oxygen tension under routine ICU monitoring conditions, where S_p_O_2_ is often used as the primary guide for oxygen titration.

The probability-based contour analyses integrate these observations by illustrating how S_p_O_2_ and F_i_O_2_ jointly determine the likelihood of exceeding predefined P_a_O_2_ thresholds (Figure 5). Rather than identifying single cutoffs, these analyses delineate contiguous regions of low and high probability across the S_p_O_2_-F_i_O_2_ plane. Small increments in S_p_O_2_ within the upper saturation range were associated with steep increases in hyperoxemia probability, particularly at moderate to high F_i_O_2_ levels. Stratification by pH demonstrated systematic shifts in these probability surfaces, further highlighting that identical saturation targets may correspond to markedly different arterial oxygen exposures depending on physiological context (Figures S1 and S2). The F_i_O_2_ ranges summarized in Table 2 are derived directly from these probability surfaces and should be interpreted as exposure-associated ranges rather than prescriptive targets.

An important and potentially counterintuitive finding concerns the relationship between respiratory failure severity and hyperoxemia. Patients without ARDS or with mild ARDS most frequently exceeded hyperoxemia thresholds, whereas patients with severe ARDS rarely achieved supraphysiological P_a_O_2_ values despite high F_i_O_2_ exposure (Figure 6). This pattern reflects profound diffusion limitation in severe ARDS rather than reduced oxygen delivery intensity. Consequently, hyperoxemia should not be interpreted as a marker of disease severity; instead, it appears most likely in patients with relatively preserved gas exchange who are exposed to liberal oxygenation strategies. This observation helps explain why hyperoxemia risk plateaued or declined at very high F_i_O_2_ levels in the overall cohort and underscores the importance of considering respiratory severity when interpreting arterial oxygen exposure (Figure 5).

Age had only a minor influence on arterial oxygen tension in patients without supplemental oxygen, with P_a_O_2_ declining less than predicted by classical reference equations [20] (Figure S3 in the Supplementary Materials). Although aging is associated with impaired alveolar diffusion [21,22,23], P_a_O_2_ values across age groups in the present analysis remained well below the predefined hyperoxemia thresholds, supporting the appropriateness of using fixed P_a_O_2_ cutoffs. These findings suggest that age-specific adjustments are unlikely to meaningfully improve the identification of hyperoxemia or the interpretation of arterial oxygen exposure in routine ICU practice.

Our findings should be viewed in the context of prior randomized controlled trials evaluating different oxygenation strategies [3,13,14,15,16]. Most of these trials compared fixed saturation or P_a_O_2_ targets and assessed clinical outcomes, with mixed or inconclusive results. In contrast, our study does not evaluate treatment strategies or outcomes but characterizes physiological oxygen exposure under routine care. By focusing on hyperoxemia as an exposure rather than on oxygen targets as interventions, our analysis provides complementary information that may help interpret why outcome differences have been difficult to demonstrate in heterogeneous patient populations.

It is important to emphasize that elevated oxygen saturation does not inherently imply oxidative injury. Oxidative stress is thought to arise when increases in dissolved arterial oxygen exceed physiological buffering capacity, rather than from hemoglobin-bound oxygen itself [5,24,25,26,27]. The absence of outcome differences in trials comparing P_a_O_2_ within physiological ranges reinforces the rationale for making this distinction [14,28]. Our study did not measure biomarkers of oxidative stress or tissue injury; therefore, mechanistic implications remain inferential and are based on established physiological and experimental evidence [17]. The present findings should thus be interpreted as identifying conditions associated with increased arterial oxygen exposure rather than demonstrating downstream biological effects.

Several limitations merit consideration. The retrospective design precludes causal inference, and oxygen delivery practices may reflect institution-specific routines. Arterial blood gases were obtained intermittently, and although careful temporal alignment was performed, short-lived fluctuations in oxygenation may not be fully captured. Our analysis addresses systemic arterial oxygen exposure and does not capture local intra-alveolar oxygen toxicity in severe ARDS [29]. The selection of thresholds was guided by the existing literature. Currently, no standardized cutoff values have been established for degrees of hyperoxemia, and there is considerable variability across studies [30]. For this reason and based on physiological rationale and previous publications [2,17,18] hyperoxemia was arbitrarily defined as mild and moderate when P_a_O_2_ exceeded 120 mmHg and 150 mmHg, respectively. Additionally, the study population was predominantly Caucasian, which limits the generalizability of our findings regarding S_p_O_2_ accuracy in individuals with darker skin pigmentation [31]. Although oxidative stress is mechanistically linked to hyperoxemia [25,32,33,34,35], our results, derived from an observational dataset, should be interpreted as reflecting physiological plausibility rather than direct causality. Finally, although both medical and surgical patients were included, detailed analyses focused on mechanically ventilated patients, in whom hyperoxemia was most prevalent.

## 5. Conclusions

Hyperoxemia is common in critically ill patients and is promoted by higher S_p_O_2_ targets, elevated F_i_O_2_, acid-base disturbances, and respiratory failure severity. S_p_O_2_ values above 97% substantially increase the probability of supraphysiological P_a_O_2_, whereas a target range of 96–97% is generally associated with lower arterial oxygen exposure across most conditions. Patients with preserved or moderately impaired gas exchange are most susceptible to hyperoxemia, while severe ARDS limits the attainment of supraphysiological P_a_O_2_ despite high F_i_O_2_ exposure. Together, these findings provide a physiology-based framework for interpreting oxygen exposure in daily practice and highlight the importance of considering F_i_O_2_, pH, and respiratory severity degree when titrating oxygen therapy.

## Figures and Tables

**Table 1:** Baseline demographic and clinical characteristics of the study cohort (*n* = 21,406).

Demographic Characteristics (*n* = 21,406)
Baseline characteristics of the included population
Age (mean ± standard deviation) years 62.4 ± 16.5 Age groups (years): Number patients (%) 18–40 2463 (11.5 %) 40–60 5731 (26.8 %) 60–80 10,234 (47.8 %) >80 2978 (13.9 %) Female 8068 (37.7 %) Male 13,338 (62.3 %) Baseline characteristics of patients without oxygen Therapy Age group (years) Mean P_a_O_2_ (mmHg) Number patients 18–35 106.8 150 36–50 105.1 175 51–65 99 235 66–80 101.6 352 81–100 105.2 228
Primary admission categories Number patients (%)
Cardiovascular diseases 8153 (37.4) Malignancies 3937 (18.1) Neurological disorders 1856 (8.5) Chronic respiratory disease 1791 (8.2) Trauma-related conditions 1770 (8.1) Gastrointestinal disorders 1509 (6.9) Infection 1384 (6.4) Endocrine disorders 331 (1.5) Others 1062 (4.9)
Relevant diagnoses and comorbidities
Heart failure 5339 (24.5) Acute kidney injury 5085 (23.3) Acute or chronic kidney disease 3619 (16.6) Delirium 4299 (19.7) SIRS (systemic inflammatory response syndrome) 2554 (11.7) Acute exacerbation of COPD 1792 (8.2) Metabolic Acidosis 1553 (7.1) Sepsis 1122 (5.2) Acute Respiratory distress Syndrome (ARDS) No ARDS 9290 (42.6) Mild 3344 (15.3) Moderate 4604 (21.2) Severe 4555 (21.1)

**Table 2:** F_i_O_2_ ranges associated with an absolute probability of hyperoxemia < 10%, stratified by S_p_O_2_ and pH. Values are shown separately for mild (P_a_O_2_ > 120 mmHg) and moderate (P_a_O_2_ > 150 mmHg) hyperoxemia thresholds.

Observed S_p_O_2_ (%)	pH Group	Maximum Safe F_i_O_2_ for Mild Hyperoxemia	Maximum Safe F_i_O_2_ for Moderate Hyperoxemia
94%	Alkalotic	100%	100%
	Normal	80%	90%
	Acidotic	75%	85%
95%	Alkalotic	70%	85%
	Normal	65%	75%
	Acidotic	65%	70%
96%	Alkalotic	60%	65%
	Normal	50%	60%
	Acidotic	50%	60%
97%	Alkalotic	40%	50%
	Normal	30–40%	50%
	Acidotic	30%	45%
98–100%	Alkalotic	30%	35–40%
	Normal	30%	35–40%
	Acidotic	30%	30–35%

## Data Availability

The data presented in this study are available, on reasonable request from the corresponding author, due to Swiss data protection laws.

## Supplementary Materials

The following supporting information can be downloaded at: https://www.mdpi.com/article/10.3390/antiox15020235/s1, Table S1: Flowchart of patient selection; Figure S1: Contour plots of absolute risk for mild and moderate hyperoxemia stratified by pH; Figure S2: Contour plots of absolute risk for mild and moderate hyperoxemia stratified by pH > 7.45; Figure S3: Comparison of observed P_a_O_2_ values in patients without supplemental oxygen against Sorbini’s predicted values; Figure S4: Relationship between P_a_O_2_, S_a_O_2_, and S_p_O_2_ across different pH categories.

## Abbreviations

The following abbreviations are used in this manuscript:

P_a_O_2_	arterial partial pressure of oxygen
S_p_O_2_	peripheral oxygen saturation
P_a_O_2_/F_i_O_2_ ratio	arterial oxygen tension to inspired oxygen fraction ratio
F_i_O_2_	inspired oxygen fraction
ICU	intensive care unit
P_a_CO_2_	arterial partial pressure of carbon dioxide
S_a_O_2_	arterial oxygen saturation
ARDS	acute respiratory distress syndrome
RCT	randomized controlled trials
