# Supplementary material for: Peripheral Oxygen Saturation Targets and Hyperoxemia in Critical Care: Influence of pH, FiO2, and Respiratory Failure
Source: Antioxidants (Basel). 2026 Feb 11;15(2):235. doi: 10.3390/antiox15020235 (PMC12938712; doi:10.3390/antiox15020235)

Peripheral Oxygen Saturation Targets and Hyperoxemia in Critical Care: Influence of pH, F_iO_2, and Respiratory Failure

Marcos Delgado, Robert Fritze, Matthias P. Hilty, Michael Krauthammer, Reto A. Schuepbach, Christoph Ganter and Jan Bartussek

SUPPLEMENTARY MATERIALS

Methods

Signal Alignment

Arterial blood gas analysis timestamps represent the time of measurement, not blood withdrawal. To correct for this delay (typically 1-5 minutes), each blood gas analysis was aligned with the median S_pO_2 and F_iO_2 values recorded during the five minutes preceding the blood gas analysis timestamp. This approach minimized bias from transient fluctuations in oxygen delivery or perfusion.

Data Quality Control

Implausible values were excluded, such as SpO_2 readings <50% or >100%, or PaO_2 >600 mmHg in the absence of extracorporeal support. ECMO patients were excluded due to altered oxygen dynamics. Artifacts in blood gas samples (e.g., air bubble contamination, implausibly high P_aO_2 on room air) were identified using distributional outlier detection and removed. Duplicate or incomplete records were excluded after cross-checking with raw logs. A summary of excluded samples and reasons is provided in Supplementary Table S1.

Additional Analyses

1. Age-related oxygenation

Patients without supplemental oxygen served as a physiological reference group. Observed P_aO_2 values were compared with Sorbini's age-adjusted predictions to assess the applicability of healthy-population norms in critically ill patients.

2. ARDS severity stratification

P_aO_2 - S_pO_2 distributions were analyzed across Berlin-defined ARDS categories. Contour plots illustrated how the risk of exceeding hyperoxemia thresholds shifted with disease severity.

3. Statistical visualizations

Histograms and cumulative distributions were used for P_aO_2 and F_iO_2 patterns. The contour plots of absolute probability were generated to display the probability of mild and moderate hyperoxemia across S_pO_2 - F_iO_2 strata, stratified by pH category. Contour levels were delineated in 10% increments of absolute risk probability. Additional subgroup analyses: Separate contour plots were created for acidotic, normal, and alkalotic pH. Kernel density plots were used to analyze P_aO_2 and S_pO_2 combinations across ARDS severity groups.

4.Software

All analyses and visualizations were conducted using MATLAB R2024b (MathWorks, Natick, MA).

Supplementary Tables and Figures

Table S1. Flowchart of patient selection. Number of excluded arterial blood gas samples and patients by exclusion category.

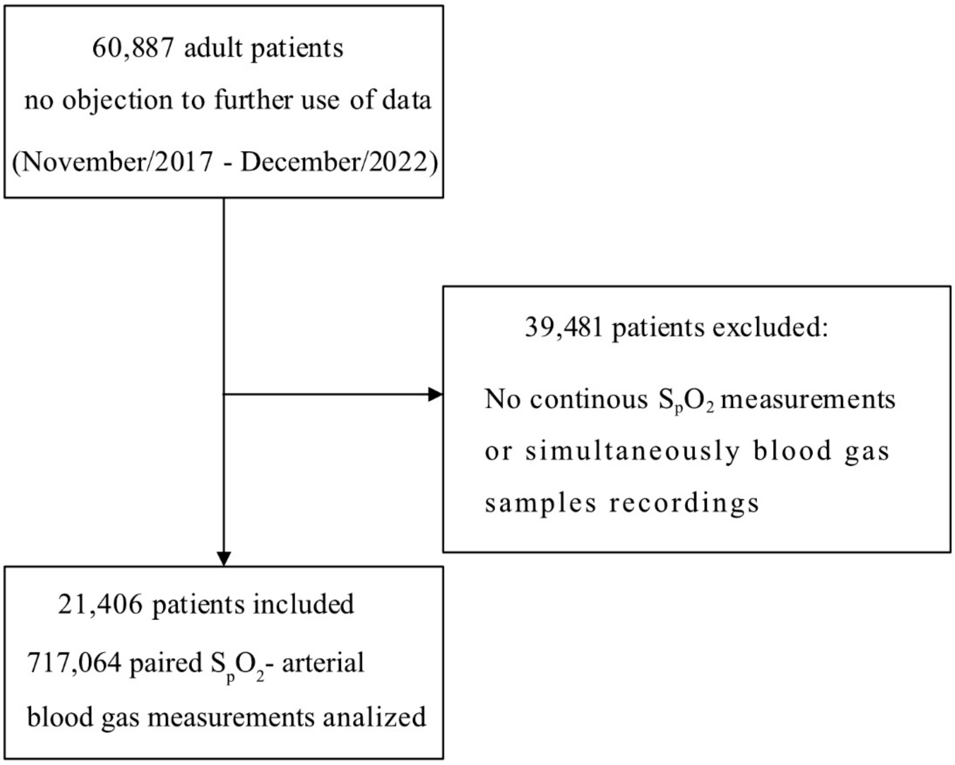

Stepwise exclusion of implausible values from the original dataset. The initial dataset contained 730,667 arterial blood gas entries from 60,887 screened patients. 21,793 individual met inclusion criteria after exclusion of patients on extracorporeal membrane oxygenation or not paired S_pO_2-blood gas analysis paired data. Sequential quality control steps eliminated (i) physiologically implausible low P_aO_2 values (likely venous samples), (ii) samples with excessive discrepancy between S_pO_2 and S_aO_2, and (iii) implausibly high P_aO_2 values without documented mechanical ventilation or oxygen therapy. After these steps, 717,064 valid entries from 21,406 patients remained available for analysis.

Figure S1. Contour plots of absolute risk for mild and moderate hyperoxemia stratified by pH <7.35. Contour plots of absolute risk of mild (P_aO_2 >120 mmHg, top) and moderate (P_aO_2 >150 mmHg, bottom) hyperoxemia in mechanically ventilated patients with low pH (<7.35). Compared with normal pH, hyperoxemia risk occurred at lower F_iO_2 and S_pO_2 values, indicating increased vulnerability in acidotic states. A plateau or decline in risk was observed at FiO_2 >80% due to the contribution of severe ARDS patients. Bins with fewer than 100 measurements were excluded to ensure stable estimates.

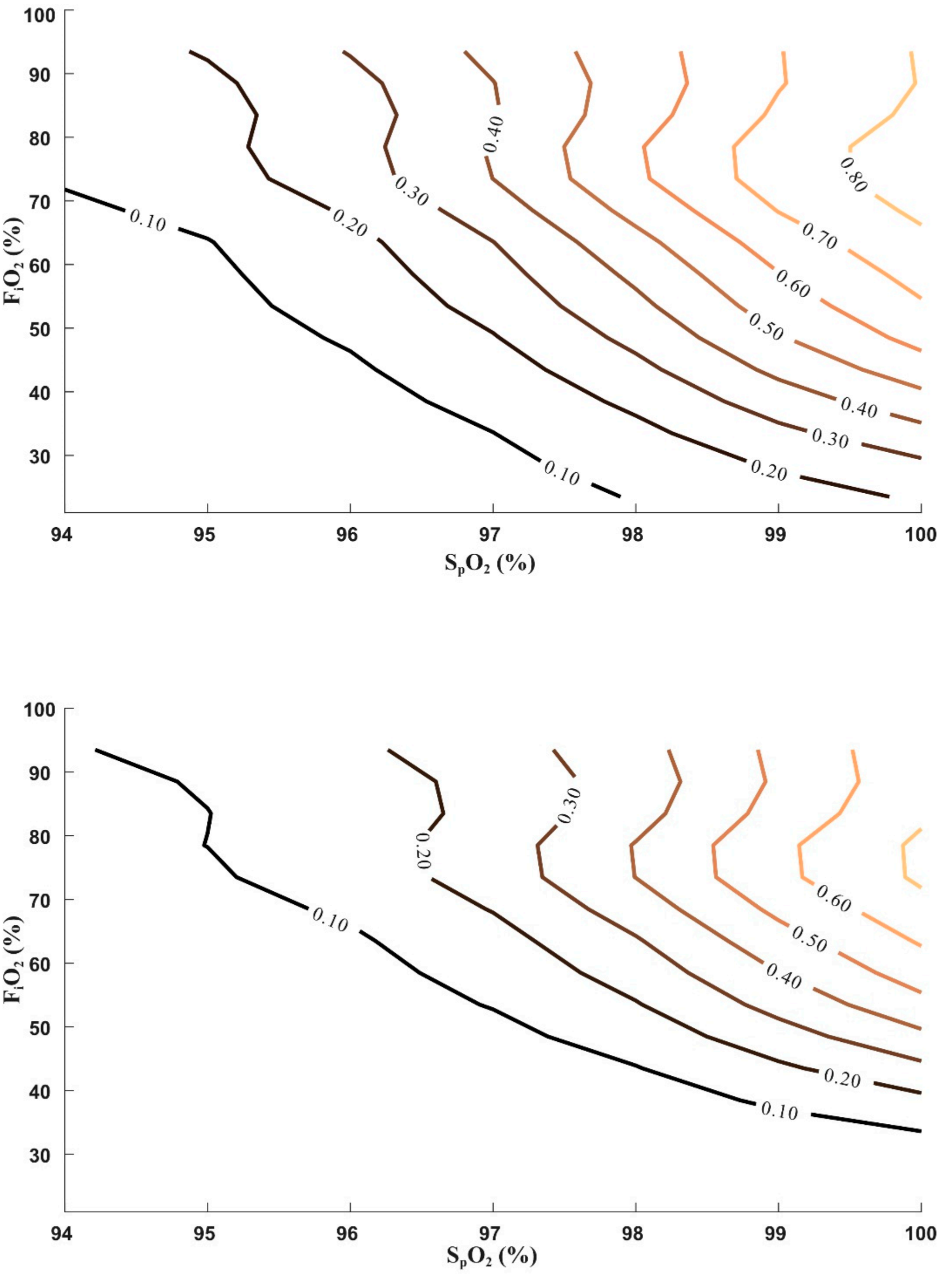

Figure S2. Contour plots of absolute risk for mild and moderate hyperoxemia stratified by pH >7.45. Contour plots of absolute risk of mild (P_aO_2 >120 mmHg, top) and moderate (P_aO_2 >150 mmHg, bottom) hyperoxemia in mechanically ventilated patients with high pH (>7.45). Compared with normal pH, hyperoxemia risk was shifted to higher F_iO_2 and S_pO_2 values, reflecting reduced vulnerability in alkalotic states. Bins with fewer than 100 measurements were excluded to ensure stable estimates.

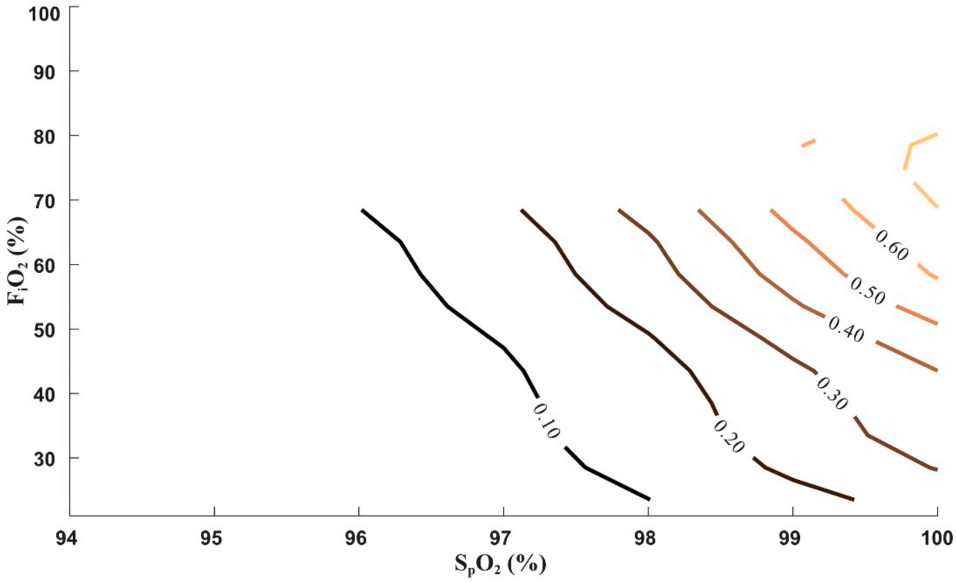

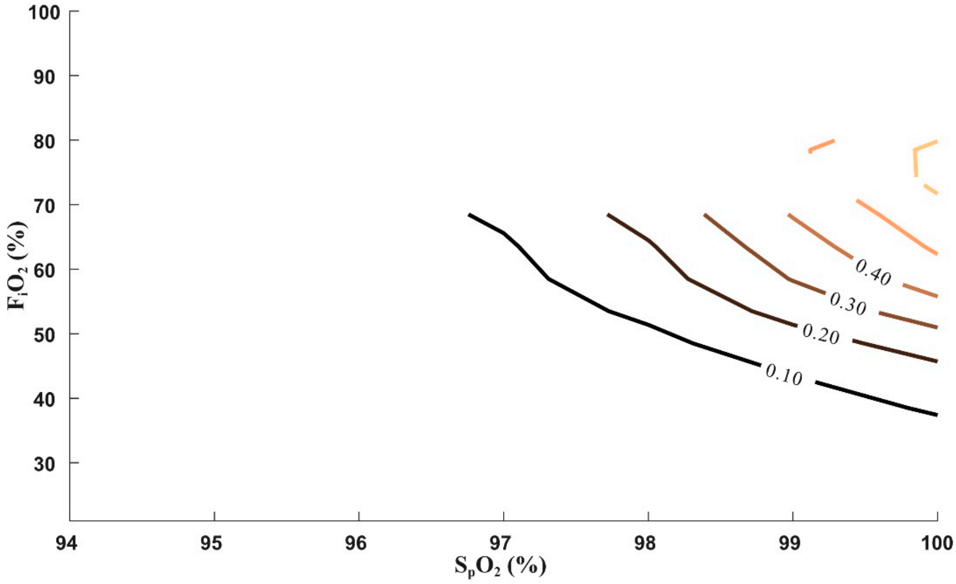

Figure S3. Comparison of observed P_aO_2 values in patients without supplemental oxygen against Sorbini's predicted values. Observed versus predicted P_aO_2 in patients without supplemental oxygen. Boxplots show P_aO_2 across age groups compared with Sorbini's predicted values. Oxygenation declined modestly with age but less than predicted, with thresholds for hyperoxemia unchanged across groups.

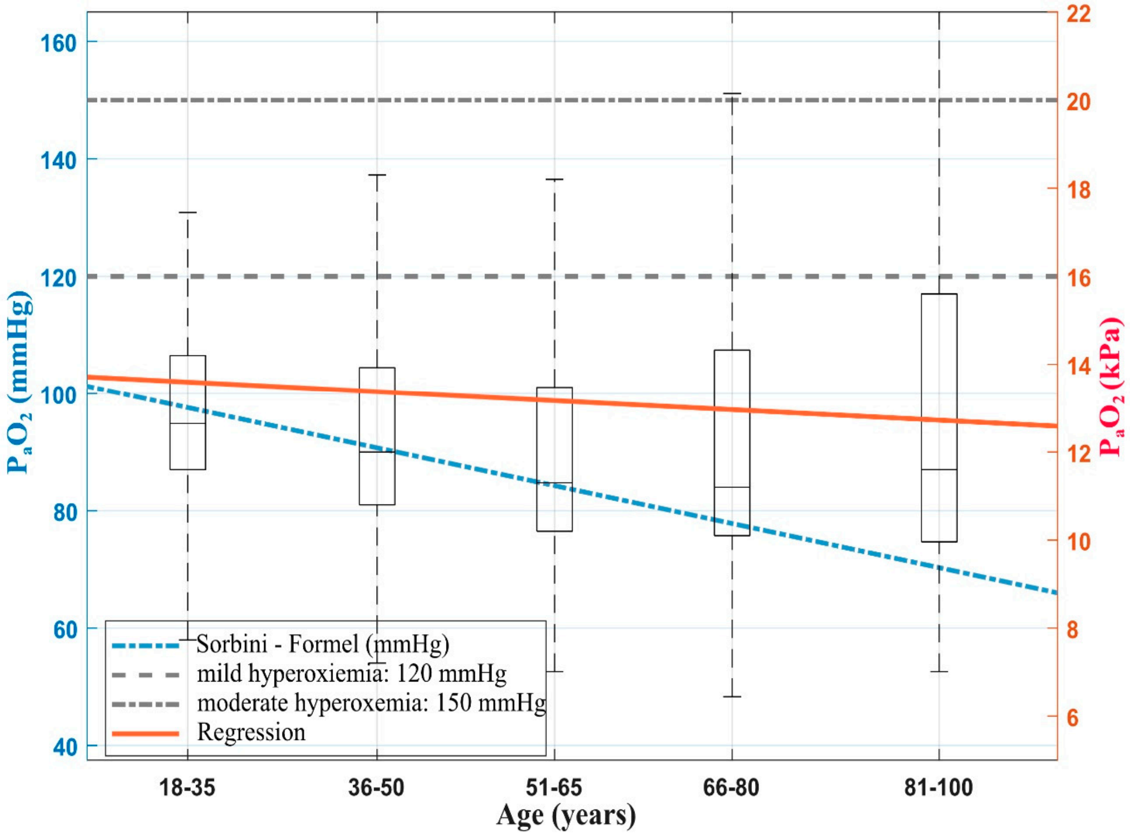

Figure S4. Relationship between P_aO_2, S_aO_2, and S_pO_2 across different pH categories. (A) P_aO_2 versus S_aO_2, and (B) P_aO_2 versus S_pO_2 demonstrate increased P_aO_2 variability at high saturation, most pronounced in acidosis. (C) Difference between S_pO_2 and S_aO_2, showing systematic S_aO_2 underestimation at high saturation levels across pH groups.

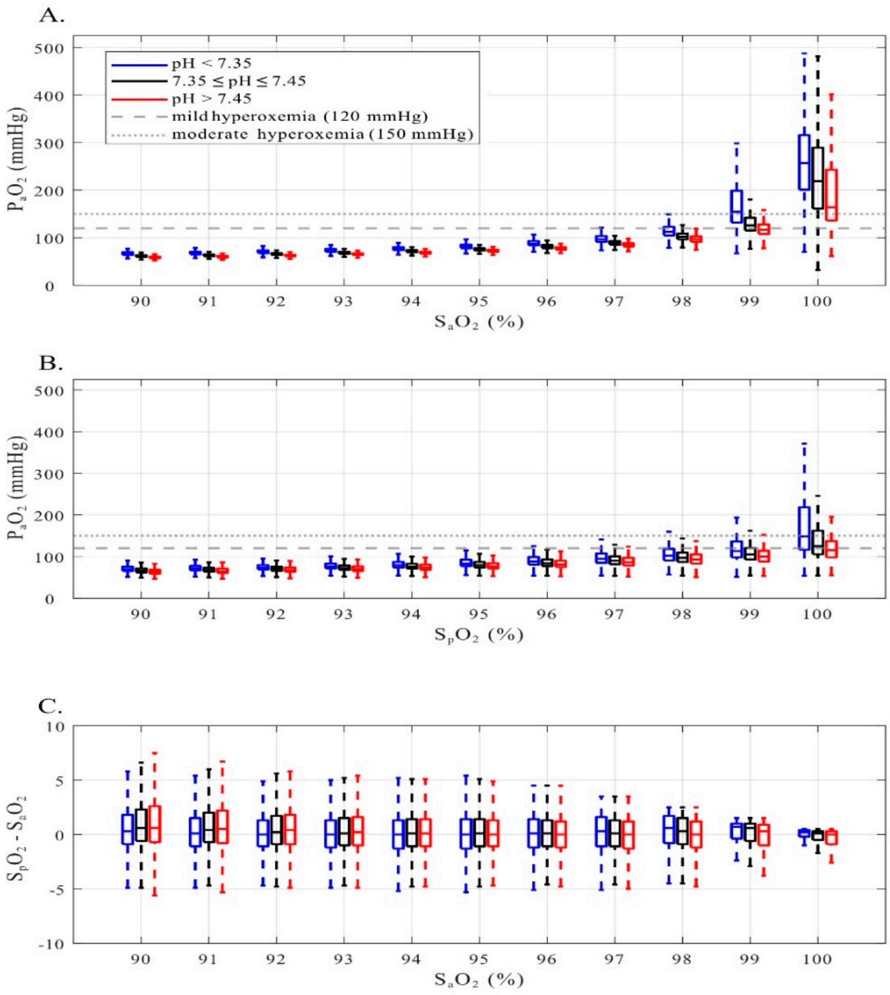

Supplement: Supplementary file 1 [file antioxidants-15-00235-s001.zip › antioxidants-4050252-supplementary.pdf]
